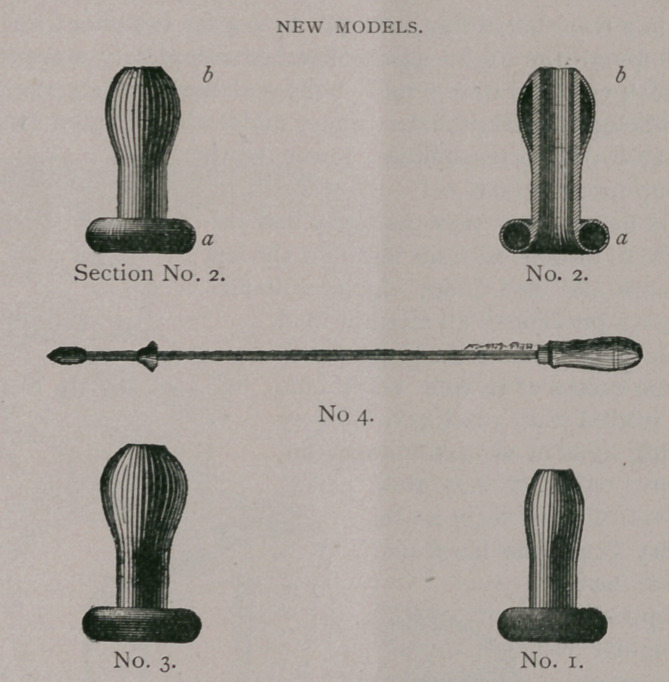# Barren Mares

**Published:** 1891-11

**Authors:** C. C. Lyford


### THE JOURNAL

OF

### COMPARATIVE MEDICINE AND VETERINARY ARCHIVES.

VOL. XII. NOVEMBER, 1891. NO. 11.

### BARREN MARES.*

C. C. LYFORD, M.D., D. V. S.

The subject of sterility, or barenness in mares, is of vastly greater importance than one would at first be led to suppose. Only those who are actually engaged in the breeding business, or are professionally called to treat such cases, can comprehend the extent, as well as the serious nature of many of these complications. Besides, in a pecuniary point of view, it is of the greatest importance to the owners of studs, as well as of mares, as very often the most valuable animals used for breeding purposes are practically of no use outside of the harem, and as a consequence, are a source of expense without any returns; when on the other hand there should be a source of revenue, often of the highest character.

Successful fecundation is generally looked upon as a sure result of coupling the male and female sex at a certain period. Fleming says: "Successful fecundation, however, is not always the case, and in some species, particularly the equine, sterility, temporary or permanent, in the female, is far from being uncommon, and is sometimes serious."

The same writer says that in the studs of France, the fruitful mares are 59.57; at the Haras of Pin, during a period of twenty years, there was a percentage of 68.27 fecund mares, abortion 5.06. This would have about 64.82 to have colts. These figures indicate that only one-half, or at the most, two-thirds of the mares produce

* Read before the United States Veterinary Medical Association, Sept. 16th, 1891.

foals. Quoting from Fleming's Obstetrics: "Sterility may depend upon organic or physical causes, and may amount to permanent impotence, more particularly when congenital, and located in the generative apparatus. Monstrosities, hermaphrodites—animals in which one or more important organs of the sexual apparatus are absent, and hybrids are generally permanently sterile."

"Prolonged continuance and old age are not infrequent causes of infecundity; as is witnessed in mares which have worked for many years in towns, and have then been transferred for breeding purposes." "It may likewise be due, though temporarily, to premature or tardy coition, when the generative organs are not in a physiological condition for conception or when they are in an irritable, abnormal state. Under-fed or over-fed animals generally do not breed so readily as those which are in moderate condition—fat animals are especially unfruitful, excitable, vicious mares, are less likely to procreate than those which are of an equable and and gentle disposition. The latter are often impregnated at one attempt; and it has been observed that with mares accustomed to work, active exertion, even to produce fatigue before being put to the horse, is favorable to conception. So it is that the Arab submits his mare to a severe gallop, and brings her almost breathless before the stallion, when, the act being accomplished, he leaves her quietly at rest for some hours."

I have known of one case where of a litter of six boar-pigs, four were fed sugar and molasses to hurry up growth, after which all four proved to be barren, while the two that were turned out on ordinary feed were productive. Again, various diseased conditions of the generative organs, as well as general derangements, may also prove antagonistic to fecundity. There may be disease or alterations in the ovaries, Fallopian tubes, uterus or vagina, which will hinder conception; and if any material obstacle to the contact of the spermatic fluid with the ovule be present in these parts, fecundation cannot take place. Tumors of various kinds in this region are not infrequent cause of sterility. Rueff and others have observed an imperforate, dense and tough hymen to be a cause of infecundity in the mare.

In all of these conditions, a careful examination should be made, as removal of the obstacle to generation may be quite within the scope of surgical or medical measures. More particularly is this the case when the obstacle is related to some abnormal condition of the cervix uteri a circumstance more common than is generally supposed. In rare instances dilation may require to be effected by a cutting instrument but this should not be resorted to until the simpler and safer means have failed.

Before taking into consideration the diseases to which the organs are subject, I will notice, briefly, the anatomy of the parts, both male and female, and their physiological functions.

MALE PELVIS (*Chauveau*)

FIG. 1.

*Anatomy of the Organs of Generation.* The penis, not only supports the greater part of the excretory, urinary canal but also transmits the sperm of the male.

The penis proper consists of the corpus cavernosum, extending to and forming the bulb, tapering gradually at the anterior extremity of the penis, occupying the upper surface and divided by a septum into two lateral halves ; grooved on its under surface for the corpus spongeosum and urethra. The corpus spongeosum encloses the urethra, extending from the crura posterior passing to the external extremity, which expands to form the glans.

A review of the anatomy can best be made by reference to Figs. 1, 2, 3, 4 and 5.

FIGURE 2.—Longitudinal Section of the free extremity of the horse's Penis in a *relaxed* state.

1, Erectile tissue of the corpus cavernosum ; 2, Urethra ; 3, Fossa navicularis ; 4, Urethral tube ; 5, Erectile tissue of the urethra ; 6, *Ditto* of the glans ; 7. Corona glandis ; 8, Urethral sinus.

FIGURE 3.—Longitudinal Section of the free extremity of the penis in an *erect* state.

1, Erectile tissue of the corpus cavernosum ; 2, Urethra ; 3, Fossa navicularis ; 4, Urethral tube ; 5, Erectile tissue of the urethra ; 6, *Ditto* of the glans ; 7, Corona glandis ; 8, Urethral sinus.

FIGURE 7.—Bladder and interpelvic portion of Urethra opened from below.

1, Vas deferens; 1′, Bulbus part of the same; 2, Peritoneal fold lining the vas deferentia; 3, Bladder; 4, Vescicula seminalis; 5, Orifices of Ureters; 6, Prostate; 7, Verum montanum with orifices of ejaculatory ducts; 8, Orifice of prostatic vesicle; 9, Cowper's Gland; 10, Orifices of ducts of prostate; 11, Orifices of ducts of Cowper's Gland; 12, Corpus cavernosum; 13, Corpus spongeosum with Urethra in its center.

FIGURE 5.—Generative organs of the mare, isolated and partly opened.

1, Ovaries; 2, Fallopian tubes; 3, Pavillion of the tube, external face; 4, *Ibid*, inner face, showing the opening in the middle; 5, Ligament of the ovary; 6, Intact horn of the uterus; 7, Horn thrown open; 8, Body of the uterus, upper face; 9, Broad Ligament; 10, Cervix, with its mucous folds; 11, *Cul de Sac* of the vagina; 12, Interior of the vagina, with its folds of mucous membrane; 13, Urinary meatus and its valve; 14, 15, Mucous fold, a vestige of the hymen; 16, Interior of the vulva; 17, Clitoris; 18, Labia of the vulva; 19, Inferior commissure of the vulva.

*Physiological Conditions of Copulation.*—It will be necessary to describe the physiological conditions of copulation to show upon what depends the normal action of the respective organs, male and female, during the act of coition. That the male organ, the penis, should be erect is necessary, and that the glans should vary considerably from their normal state is also essential. It will be seen by reference to figure, number three, that the glans and penis assumes the form of a valve and piston. The enlarged glans should fill the transverse diameter of vagina so completely as to withdraw and expel the air thus forming a vacuum within the cervix and uterus, and in case the cervix is kept sufficiently open and retained in the center of canal either by natural or artificial means so that the urethral sinus of the glans shall fit the corresponding posterior surface of the cervix, and that the projecting end of the urethral tube may approximate most closely or even fit into the opening of the os. Accordingly, should no obstruction exist between vagina and uterus, these conditions assure a complete injection of semen into the cervix and uterus, and as the glans assumes its natural size by its withdrawal from the vagina, allowing air to pass into the uterus, it practically assures the access of seminal fluid.

It is evident that a variety of influences may interfere with the performance of the natural process of fecundation. For its accomplishment, four things are necessary:

*First*, The possibility of the entrance of seminal fluid into the uterus. *Second*, The possibility of the production of a healthy ovule. *Third*, The possibility of the entrance of the ovule into the uterus. *Fourth*, The absence of influences in utero destructive to the vitality of the semen, and preventive of fixation of the ovum upon the uterine wall.

Should these four conditions exist, no animal will be sterile. She may not bear a foal, but the incapacity may attach to the male and not to her.

The special causes of sterility, or those interfering with these conditions, may thus be presented:

*First*, *Causes preventing entrance of semen into uterus: a*, Absence of the uterus or vagina; *b*, Persisting hymen; *c*, Vaginismus; *d*, Atresia vaginæ, or complete obliteration; *e*, Occlusion of cervical canal; *f*, Conical shape, elongated cervix; *g*, Patulous os and flaccid or flabby condition of uterus; *h*, Endometritis, or leucorrhoea; *i*, Polypi, or fibroids; *j*, Flexion of uterus; *k*, Very small os internum; *l*, A curtain of membrane either, or both, external or nternal to cervix; *m*, Equine syphilis.

*Second, Causes preventing the production of a healthy ovule: a* Chronic ovaritis; *b*, Cystic disease of both ovaries; *c*, Cellulitis or peritonitis, obliteration of Fallopian tubes; *d*, Absence of ovaries; *e*, Hemorrhage into ovaries; *f*, Undeveloped state of ovaries; *g*, Atrophy of ovaries from old age.

*Third, Causes preventing passage of ovum into uterus: a*, Stricture or obliteration of Fallopian Tubes; *b*, Absence of Fallopian tubes; *c*, Detachment and displacement of Fallopian tubes.

*Fourth, Causes destroying vitality of semen or preventing fixation of impregnated ovum: a*, Corporal or cervical endometritis; *b*, Membranous dysmenorrhoea; *c*, Menorrhagia or metrorrhagia; *d*, Abnormal growths; *e*, Areolar hyperplasia.

*Absence of Uterus or Vagina.*—I have met with but one case of absence of the uterus. During the summer of 1872, a young heifer showed signs of rut, and having a bull much larger than she was it was not surprising to have her look droopy after copulation, especially as she was pushed through an ordinary board-fence at that time. The heifer was allowed to stand around and attend to herself some three or four days; during which time she continually strained as if to urinate, occasionally passing a small quantity of blood. Having killed her about the fourth day I made an autopsy finding the abdominal cavity containing several gallons of urine and a hole through the anterior portion of the bladder, showing that the penis had passed through the meatus urinarius and ruptured the bladder. Two small congested ovaries were found but no uterus.

*Persisting Hymen.*—I have met with quite a number of cases of this kind and in most of these it was thicker than natural; some of the cases requiring considerable force to rupture the membrane.

*Vaginismus*, or hyperæsthesic state of the os vagina, which results in spasms of sphincter.—These cases are by no means rare, and are a common cause of sterility. It not only interferes with the entrance of the male organ, because of the pain induced, but prevents the seminal fluid from getting into the uterus, as the stallion in these cases is usually prevented making a closer cover besides the spasmodic condition completely closing the cervix.

*Atresia* of the vagina is not very common in mares, and then follows laceration, or an organization of inflammatory lymph. These conditions, however, appear very common in cows; more often following the first calf than subsequent cases. The treatment is generally unsatisfactory, requiring instruments and surgical treatment which is often of no avail. The results being anything but satisfactory.

*Occlusion of Cervix or Rigidity of Os Uteri.*—According to Fleming, "occlusion of the cervical canal may be due to spasmodic conditions of the muscle and cervix. If, however, there be hypertrophy in this organization or rigidity, then an operation will be necessary.

Both rigidity and spasmodic condition of the os uteri are of very common occurrence and are liable to be associated with many of the other troubles of the female generative organs. The spasmodic condition may be simply a consequence of irritation elsewhere. This condition is most common in young mares that have never conceived; but I have met with one case of rigidity when the mare was twenty-three years old, and was the dam of several colts. I have also met with the spasmodic condition in some cases during one heat, while at the next period it had entirely disappeared. This will, I think, account for many of the cases which have been served repeatedly during a season and all at once conceive by a single leap from another stallion or even the same one.

*Conical shape of Cervix and elongated Os Uteri* is a very common cause of infecundity. By its bending on itself it may not admit the seminal fluid through the canal, and as a rule completely prevents it. This state of affairs not only causes trouble with the breeding of mares but also in the human family. Thomas, on diseases of woman, says, "my experience leads me very positively to the conclusion that excepting endometritis, this is the most common of all causes of sterility, and fortunately remediable." "The treatment recommended varies somewhat in the human family with the length of cervix from dilation, bilateral operation and amputation." It is very apparent with these conditions in mares that the cervix does not draw down and become flat and open as it should do when the vagina "bellows up," or become rigid as it should ordinarily during copulation. For these reasons the cervix is left projecting into the vagina to the extent of two or three inches; consequently the glans penis presses it to one side during the act of copulation, and there is little or no chance for the semen to get into the uterus. When the pressure is removed the cervix projects into the vagina thus preventing the semen from entering. Right here, I will say it is not necessary for the cervix to be tense and closed, for I have known many cases where the cervix was long loose and flabby with an opening sufficiently large to admit two fingers, and still the mare fail to conceive until artificial means were used. I wish to sight but two cases here of the tense or closed os:—one, Belvedere by Mambrino Patchen, another, Gypsey Queen by Polanios—and one case of elongated, patulous cervix in a mare of my own, Mabil H, 2:26, by Col. West, 2579. In regard to the first two cases, I will quote from the letters received concerning them. Byron G. Kimball, of Maple Stock Farm, Bradford, Mass., says: "The mare, Belvedere, I bought of William Trumbull, of New York City, for Col. H. A. Hale of Bradford, and sold her at a sale in Boston, where she was bought by W. H. Phelps of Minneapolis. I had bred this mare according to my books on an average of twice a month for twenty-seven months with Warder Hudson and various other stallions. I tried an impregnator on her but it did no good. It was rubber but more bell-shaped than yours." Dr. O. J. Evans, of Evansdale Stock Farm, Minneapolis, Minn., says: "Having used your impregnator on my Membrino Patchen mare Belvedere, nineteen years old, that was bred by Mr. Henry Hale, of Bradford, Mass., to Warder by Belmont and to Hudson by Kentucky Prince, and by H.W. Phelps of Minneapolis, Minn., to Bayardo at least four times all without impregnation, and having succeeded in getting her in foal during first heat by Red Chieftain using the impregnator, and having used it on several other mares that had refused to breed one or more seasons, among them, Gypsy Queen, by Polanius, she being a mare, twelve years old, and had been bred to different horses at least four seasons without becoming in foal. I must also state that neither Belvedere or Gypsy Queen had ever been in foal until this season and both are now sure."

F. W. Muckey, Minneapolis, Minn., says, "I owned the bay mare Gypsy Queen, and bred her two years without success. I then sold her to J. K. Sidall, of Minneapolis, thinking her barren, as she was a young mare and we had used every means then known to the profession. Since then she has become the property of Dr. O. J. Evans, and I understand he has been unsuccessful until he used your "Impregnator," and with the first trial succeeded in getting her in foal." In regard to the case of elongated patulous cervix in Mabel H., she was a mare who at the age of five years had had a filly by Phallas 2.13¾. The next two years she was not stinted but was returned to Phallas for the season of 1887 and 1888, but failed to conceive. In February 1889 she was sent to T. B. Merritt's Farm at Rosemont, Minn., and was stinted to Nutwood Membrino until June 12th, without any good results. June 13th, 1889, I again had her returned to Nutwood Membrino using an Impregnator from which she conceived; the result being a chestnut foal, born June 13th, 1890, now registered Vol. X. Wallace Trotting Register as Woodnot 15234.

*Patulous Os or flabby condition of the Uterus.*—These cases are very common in mares, generally in those which have had foals or aborted, but are sometimes seen in mares which have never been in foal, or even stinted. The cervix is very loose and flabby, which is often more or less associated with a like condition of vagina and uterus; the os at times being so open as to admit the entire hand with little or no resistance. I had a cases of this kind at the Bruce Stock Farm, Rosemont, Minn. The mare had aborted over a year before, since which time they were unable to get her in foal. She appeared otherwise in good health, worked every day and kept in good heart and flesh. I had the uterus and vagina flooded daily for five weeks using for an injection, alternate days, carbolic acid 1—100 warm water, corrosive sublimate 1—1000. This was continued until signs of heat returned the second time, when after being stopped her cervix was swabed out with iodoform ointment 1—10 and the third day was served. The os had so contracted that the large sized impregnator went in with difficulty though the dilater was used. When examined before treatment her cervix would admit with ease the entire hand; the uterus and vagina being especially flabby. The mare has since failed to receive another embrace though repeatedly tried for over three months and shows every indication of being in foal.

Another case of this kind was one of my own, Nellie Gray, Dam of Mabel H.; a mare twenty years old, having failed to conceive for five years—and having aborted six years ago—though being repeatedly stinted to various stallions before I purchased her in 1889. I had her stinted during the season of 1889 to Col. West, 2579, and during the season of 1890 to Morrel Tyrant and Greymont, the last two being young stallions, but to no avail. During the fall of 1890 I examined her, finding the cervix not only sufficiently open to admit easily three fingers, but the cervix was torn on its upper portion and on the right side of *cul-de-sac* of the vagina from the vagina wall to cervix was a complete honeycomb; having evidently been lacerated at various times during copulation. Having decided to give her tonic treatment and regular exercise, she was left without further stinting until April 1891, when I examined her and found the vagina and cervix nicely contracted and in heat. She was then stinted to a three year old son of Jersey Wilkes, from which she is now surely in foal.

Various modes of treatment have been tried for the lax, weakened condition of cervix and uterus, which may be classed as constitutional and local. The former class of remedies I have not given a thorough trial, though the cases on which I have used them indicate favorable results. These consist of general tonics and especially stimulating and invigorating aphrodisiacs; such as phosphorus, cannabis indicus, nux vomica, ergot and arsenate of iron. Also saw palmetto Fld. Ext.

Local treatment, such as swabing cervix with tr. iodine and iodoform, as well as stiptic, astringent, and antiseptic injections have apparently proven beneficial in a number of cases.

I believe that electricity will prove itself very useful in these cases; especially where applied locally to the cervix, vagina or uterus.

*Endometritis* fills the uterine canal with a thick tenaceous mucus and often prevents the entrance of seminal fluid or destroys its vitality. We meet with quite a good many of these cases in the mare, and they vary very materially in the consistency of the secretions. Endometritis and resulting leucorrhoea are the most unsatisfactory diseases we have to contend with in the treatment of barreness. In the first place it is far from being an agreeable task, and as the cases are generally of long standing when we get them, they are not only the more difficult to cure but the time and expense often exceeds the value of the animal. The mare as a rule is emaciated; cannot stand hard work, and, though her appetite is often good, fails to put on flesh. The discharge is of a viscid, glassy or creamy character—often with a peculiar odor, which we require to smell but once to remember; especially in every case you get to attend at college, when you have to depend upon your fellow-students to assist you in treatment, particularly the injections, the smell stays by you often for a day or more, no matter how often you wash or use disinfectants. I am glad to say that the balance of my cases have been looked after by the owners or persons in charge, though it is often a great deal of trouble to get them to follow your instructions, and get anything like favorable results. Mineral and vegetable tonics and mineral acids have generally proven beneficial. Antiseptic injections, not too strong, also perox. hydrogen; as there is some danger of overdoing. Unless the os is flascid and well dilated it is better to keep the parts open to allow drainage; as

I have known of cases when fluid was retained from one day to the next, the horns of the uterus often being relaxed.

*Polypi, fibroids and moles* are not very common in my experience; having met with but three cases, all of them being outside of cervix, and were very easily cured by excision, stiptic and antiseptic dressings.

*Flexion of uterus and cervix* is not uncommon. In this the os is turned to one side and during copulation it would be pressed against the wall of the vagina, entirely obstructing the passage to the uterus. Huntress 2.21 is said to be one of this kind; having been examined by R. C. Mason, V. S., of Winona, Minn., who reported the case to me as such a decided flexion that he was compelled to turn his finger almost at a right angle to get through the cervix.

*Very small os internum.* It is a common thing to find barren mares who have been continually bred and repeatedly opened by breeders, stablemen and even veterinary surgeons, without the inner portion of the os being dilated, and at other times a membrane across the os internum which is not ruptured. As a consequence they fail to conceive as effectually as if the membrane were over the vaginal surface of cervix.

*A curtain of membrane, either or both, external or internal to cervix.*—A very interesting case of this type came under my treatment during the month of July, 1891. The mare was sixteen years old, and had failed to conceive, though stinted repeatedly at different seasons for the past ten years. I had known the mare some six years, she having been served by one of my own stallions during the year of 1886 but had given the case no special attention, and at that time knew nothing more than that she was claimed to be very tight by the man who dilated her os. She was given several leaps but did not conceive, and she was stinted every season following to different stallions but to no purpose. She was then sent to Dr. Curryer & Sons' stud at Crystal Lake, Minn., with instructions to use the impregnator. The Doctor was unable to find the os uteri; it being concealed by folds of mucous membrane. I was called to examine the case and found a fold of membrane reflected from upper vaginal surface of the os. Having passed one finger underneath the fold of membrane with a good deal of difficulty, I succeeded in dilating it sufficiently to get one finger through the cervix. I could then easily feel a second membrane at internal opening of cervix, but my finger not being long enough or the membrane was so strong that I could not tear it. By taking a small impregnator and dilator which is about one inch longer than my finger and passing it through the cervix until the disk of impregnator came in contact with the vaginal surface of cervix, I then made a thrust by pressure to handle of dilator at the same time turning it laterally. I then withdrew the dilator leaving the impregnator in position. The mare, at once, by straining, threw off at least three pints of viscid, creamy fluid, which had no odor. I then had the uterus washed out, which was continued daily until appearance of heat returned some two weeks later. She was then served, using the small impregnator, and has since passed three periods or about six weeks, having been tried twice a week without any signs of returning heat. I simply wish to call your attention to the facts concerning this case: The mare had been repeatedly opened by parties who would generally be considered competent judges and capable of opening mares to breed. This mare had been so treated by several such men besides having been examined by a graduate veterinary surgeon, who also used the small sized impregnator, having succeeded in placing it without the dilator. The external fold of membrane was reported, but the internal one was not noticed, and not being ruptured, there could be no chance of conception so long as it existed.

*Equine syphilis* has proven a great hinderance to breeding by rendering pregnancy both uncertain and unsafe, and requires especial consideration for which I would refer to W. L. William's article on equine syphilis in *American Veterinary Review*, 1888.

CLASS II —*Causes preventing the Production of a Healthy Ovum.*—I will notice but one the atrophy of ovum from old age and lack of use, as it will be seen by reference that none of these are curable diseases. I wish to note but one case, that of a black mare, record 2:53¼, belonging to me; I having bought her in 1887 to experiment on. She had never had a foal though bred several seasons. After various trials, even by injecting semen through the cervix, she continued to return in heat, and in December, I decided to kill her and hold an autopsy. The uterus, vagina and cervix were healthy and in every way normal, but on examination of ovaries, they were found to be pale and atrophied; showing no signs of graafian vesicles, or any indication of having produced any ovum for months, possibly for years.

III.—*Causes preventing Passage of Ovum into Uterus*, such as strictures or obliteration of Fallopian tubes, absence of Fallopian tubes, detachments and displacements, simply require mentioning to show how certainly they would prevent conception.

IV.—*Causes destroying Vitality of Semen or preventing Fixation of impregnated Ovum.*

*a.* Endometritis, corporal or cervical, fills the uterine canal with mucus which either prevents the entrance of semen, or destroys its vitality, and has already been considered.

*b.* Abnormal growths of any kind which fill the uterine cavity, as for example fibroids, polypi, etc., may prevent attachments of the ovum to the uterus even if impregnated.

*c.* Membranous dysmenorrhagia, menorrhagia or metorrhagia and areolar hyperplasia are seldom if ever seen in mares, hence will be given no further consideration.

MALE STERILITY.—Lack of erectile power in the male is not uncommon and varies with different stallions as well as the same stallion at different seasons, or portions of the same. At the beginning of the stud season many stallions fail to perform service with sufficient ardor, although they have been good coverers seasons previous. This may be caused by lack of tone from non-use, though at other times such a state of things follows certain diseases; such as catarrhal fevers, distemper and the like, as well attacks as of spinal meningitis. In other cases the blood supply may be interfered with from partial or complete obstruction to one or more of the arteries supplying the penis.

During the spring of 1886, a stallion was brought to my infirmary with apparent paralysis of the penis; the parts hanging pendulous, protruding about six inches. The season previous he had covered about sixty mares, and had gone into winter quarters in good shape, but during the winter suffered from an attack of catarrhal fever, during which time his owner reported him badly swollen about the penis and testes, after which penis remained pendulous. The stallion had already been blistered across the back several times. I applied electricity to the parts, which would at the time produce partial erection and so strengthen them as to enable him to withdraw within the sheath, but he never regained power of erection or afterward performed stud service.

During the Spring of 1889, I was consulted in regard to a stallion, who, the previous season, had served forty mares, and was sold with a warranty of a sure foal getter; but, as he would not cover a mare at the beginning of the stud season, the party purchasing naturally suspected he had been cheated. I recommended as treatment:—Fl. Ext. Nux Vomica, Liq. Pot. arsenitis, Fl. Ext. ergot, and citrate of Iron, alternating with phosphide of zinc and sanguinaria. This treatment was continued but a short time when the animal's vigor returned, and there was no further trouble that season. I also had a case of my own, a four year old stallion, who had been a good coverer until the Fall of 1888, when I loaned him to cover some mares in the country, at which time he was kicked in the front leg and nearly died as a consequence of erysipelas and distemper which followed. The following season I could scarcely get him to cover a mare, and then invariably failed to get them in foal until the foregoing prescription was used, when he succeeded in getting all five mares in foal.

Absence of spermatozoa is not uncommon especially in colts less than two years old, and as a rule at any age, should the testicles not appear in their natural locality, the scrotum. In cryptorchids, as a rule, when neither of the testicles appear visible, no spermatozoa are to be found.

Old age is a common cause of impotency, but a great deal can be done to tone up these organs, and revive the natural functions by judicial use of some of the remedies which prove so beneficial in the lack of erectile powers.

Excessive length of penis is far from being an advantage either to male or female. Such stallions are seldom sure foal getters, and often injure the mare during copulation. I have found in these cases great advantage in using a shoe-boil boot as a washer, thus keeping six or eight inches of penis outside the vagina, and in many cases, it has insured foals, where the stallion was considered not only unsafe to the mare but uncertain as a foal getter, On the contrary, stallions with a short penis will cover a greater number of mares, and succeed in larger percentage of foals.

During the Summer of 1882, I stinted two mares to Seneca Starr; he was a large horse with excessive length of penis and a very ardent coverer, though apparently not a sure foal getter. Having previously injured several mares and killing one by lacerating the fundus of vagina, I decided to try one by using a shoeboil pad as a washer. The move succeeded in getting her in foal at the first service. The other mare was stinted without making use of the pad, and though returned several times, did not get in foal, though she had been a regular breeder before and had a colt by her side. The only mare in foal to Seneca Starr that season was the one on which the pad was used. The next season the pad was made use of in serving mares to him and as a result he got some twenty mares in foal.

Weakness of Spermatoza —There is little doubt that the vitality of spermatozoa differs very materially in different stallions as well as in different kinds of animals. I have at various times examined spermatozoa under the microscope from different stallions after castration, as a rule, having a pail of water in which to place the testicles after removing them. By so doing they were all kept as nearly as possible under the same atmospheric conditions. The only difference being the length of time between subsequent castrations. When ready to make microscopic examination of semen, I would lay the different sets of testicles by themselves outside the water, and put on a glass slide, under a top cover, a specimen from each set, and examine them at different intervals of fifteen minutes to one-half an hour. During the Summer of 1877, I made a number of these experiments, and invariably found the spermatozoa from one set of testicles would outlive the others, and as a rule, those stallions whose testicles showed signs of injury, or inflammatory process showed less vitality; whereas the size of the testicle seemed to make little difference with the vitality of semen, both being healthy. Small or medium sized testicles, as a rule, are less subject to injury, especially in stallions that are tracked or given fast road work. In one case particularly where specimens were examined, the animals having been castrated between eight and nine o'clock in the morning on a moist warm summer day, the specimens were prepared and examined between nine and half past nine in the morning. I had occasion to show these to parties as late as five in the afternoon of the same day, and, to my surprise, the specimen from one set of testicles still showed vitality enough to move, while all the others showed no signs whatever of life.

I am of the belief that under favorable circumstances, if properly prepared, the semen of a stallion can be kept for several days; and that, at some future date, we will be able to send specimens of semen to be injected, instead of mares to be served. This would not only save the expense and time of shipping the mare, but a single service of a valuable stallion could be used to impregnate a number of mares, by which means a stallion could as easily get two hundred colts each season as fifty by ordinary methods.

### The Different Instruments and Remedies now Advertised for Barrenness in Mares.

I present, for your consideration, these instruments, and principles indicating, ideas of greater or less value; but all point towards one great principle—the dilation of the cervix and its retention in that position and centre of canal. To say that any one or all of the instruments can prove successful in every case, is an impossibility, though I am sorry that such advertisements as the following are to be found in our stock papers regarding one of them at least—"Barren mares made to breed regularly. *All* mares made to conceive at first service." This makes it practically non-professional, and is a poor recommendation for parties who indorse such statements. It may serve a special purpose in certain cases, but it is far from being infallible, and its claim is not only unjust and misleading to breeders but unreasonable and erroneous. The Eureka requires from six to ten hours for expansion, hence the mare must either wait or return for service the next day. It is the most expensive one of the kind in the market as each service requires a new instrument.

The funnel shape instrument is practically out of use on account of the great difficulty attached in placing it. The exceeding wide spreading end that is intended to pass through the cervix has to be folded or rolled very tightly in order to get it even in a fairly loose os; and in cases that are at all constricted, less than to admit two fingers, it serves little or no purpose. Even should it be crowded into the cervix, it cannot expand, and is either thrown out by the mare, or works out during the act of copulation.

The Meddick pattern consists of a flat disk and a soft rubber tube; the latter surrounded by convolutions or flanges of rubber to retain it in position, and is held in shape by a hard rubber tube, small enough to pass through the others. This is too complicated to be practical, even if the convolutions were not a source of annoyance in removing it from the cervix, and in retaining filth unless *every precaution* in cleansing and disinfecting is followed after each service. Besides, this is a hard rubber tube, when the least projecting, would subject the glans penis to more or less pain (if not injury) in proportion to the closeness of the cover and the ardor and impetuosity of the stallion.

As to medical remedies, those kindly sent by Mr. Wallace Banus for your inspection can not be given a professional standing on account of the misnomers, under which they are to be recognized; not being represented either in Allopathic or Homœopathic medicines ; consequently are shrouded in mystery, which injures their reputation at least from the professional standpoint.

As to my own patterns of Impregnators and Dilators, Figures I, II, III, represent those which have been in general use for several years. The impregnators consist of a hollow tube or cone

I.

II.

OLD MODELS.

III.

composed of soft rubber of sufficient thickness and firmness to retain its shape and resist the pressure of cervix. Somewhat constricted at the disk portion that it may be self-retaining, the disk on the posterior surface is made so as to correspond to the urethral sinus of the glans, while the opening through the disk is sufficiently large to admit the projecting end of urethral tube. The greatest difficulty is to make the two sizes meet all of the requirements and variations of the cervix, as well as the peculiararities of the stallion, and the idiosyncrasies of the owner or attendant. In certain cases No. I (small size) proves difficult to insert on account of the close tense os, but with the dilator this is quite easily obviated. In other cases No. II (larger size) may be too small to be retained and requires a larger size.

Some stallions are especially sensitive while covering a mare —generally those stallions whose parts are larger than normal or those having a big season, and are not very anxious when they find the least interferance. To obviate these difficulties I have to present you the new models of impregnators and dilaters represented by cuts Nos. 1, 2, section No. 2, No. 3 and No. 4. They consist of the same size tube internally, so that a single dilator fits the entire set, while the external dimentions correspond to the size of the cervix any where from an inch to two and one-fourth inches in diameter and from three and one-half to four and one-half inches in length. By section of No. 2, it will be seen that the disk (*a*)

NEW MODELS.

Section No. 2.

No. 2.

No 4.

No. 3.

No. 1.

consists of a hollow air space as well as the bulb (*b*). The disk closely corresponds to the os in pliability, and the most sensitive stallions should fail to perceive the difference and as a consequence make an equally close cover as when no instrument is used. The advantages in favor of the tubular variety of inpregnators, are the close approximation to the normal condition of the cervix during heat, rendering complete—as it does—the communication between vagina and uterus, thus assuring easy access for the seminal fluid, besides being easily inserted and ready for immediate use. They are *cheap* as one *will last for years*, and can be used on any number of cases.

## Figures and Tables

**Fig. 1. f1:**
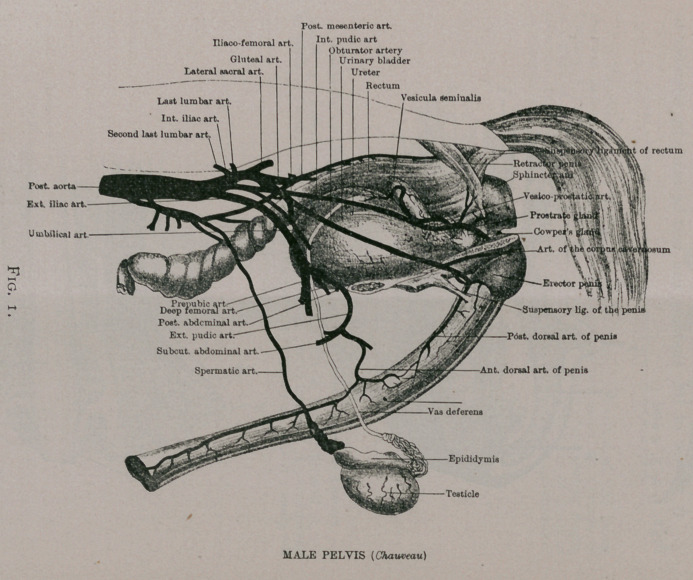


**Figure 2. f2:**
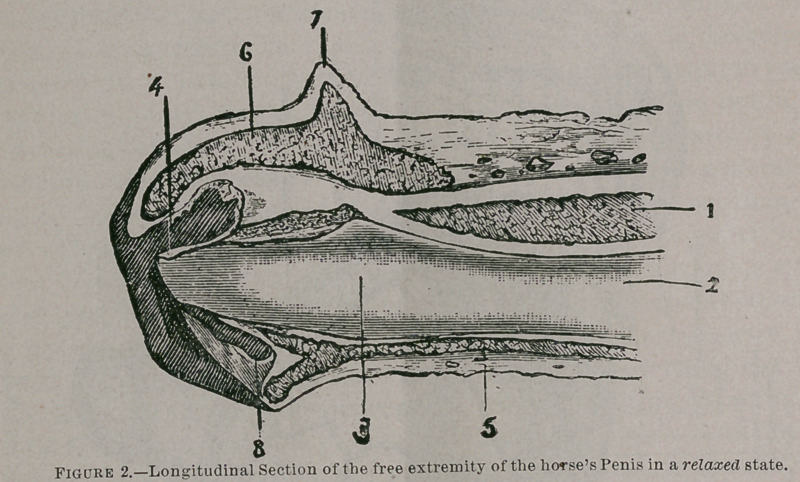


**Figure 3. f3:**
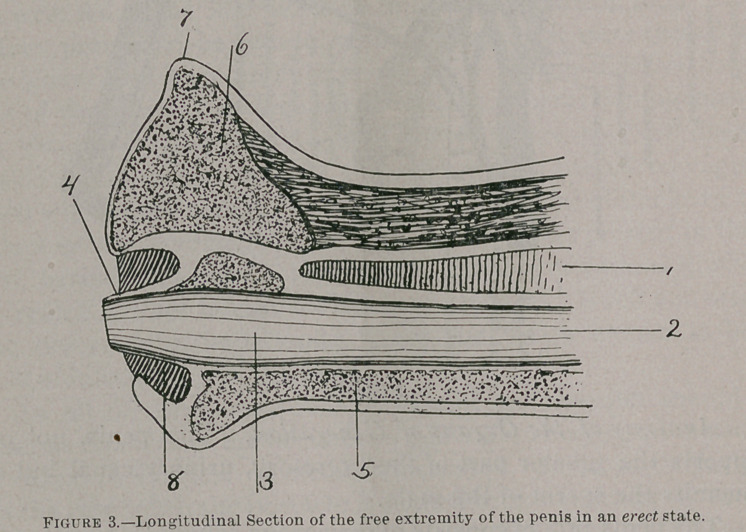


**Figure 7. f4:**
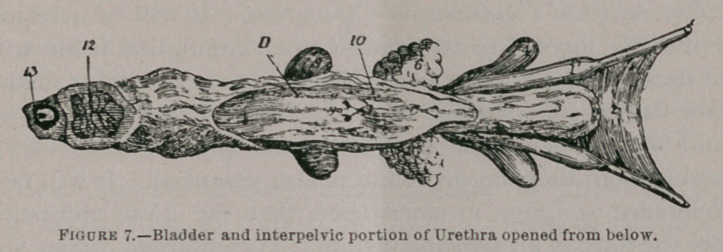


**Figure 5. f5:**
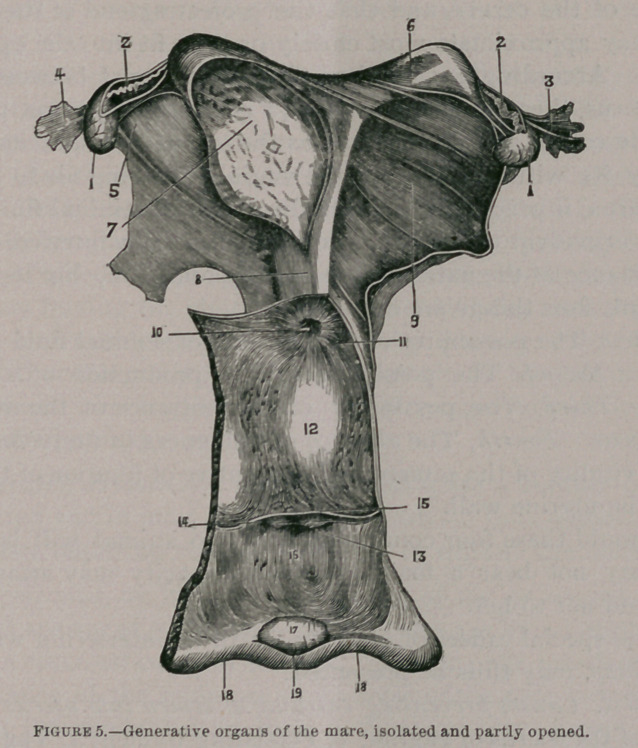


**Figure f6:**
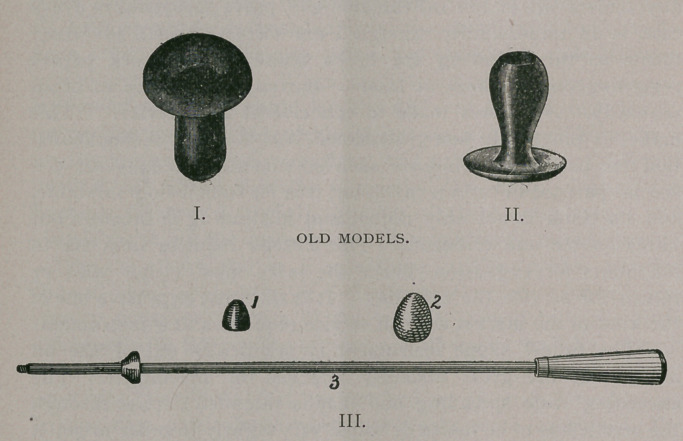


**Figure f7:**